# A short history of the successes and failures of the international climate change negotiations

**DOI:** 10.14324/111.444/ucloe.000059

**Published:** 2023-07-19

**Authors:** Mark A. Maslin, John Lang, Fiona Harvey

**Affiliations:** 1 Department of Geography, University College London, North-West Wing, Gower Street, London, WC1E 6BT, UK; 2 Natural History Museum of Denmark, University of Copenhagen, Gothersgade 130, 1123 København K, Denmark; 3 Energy and Climate Intelligence Unit, 180 Borough High Street, London, SE1 1LB, UK; 4 Consult Climate, 48 Caistor Road, Balham, London, SW12 8PZ, UK; 5 The Guardian, Kings Place, 90 York Way, London, N1 9GU, UK

**Keywords:** climate change, negotiations, UNFCCC, COP26, COP27, Paris Agreement, Kyoto Protocol, net zero, climate emergency, environmental social movements

## Abstract

The last 35 years have been a period of intense and continuous international negotiations to deal with climate change. During the same period of time humanity has doubled the amount of anthropogenic carbon dioxide in the atmosphere. There has, however, been progress and some notable successes in the negotiations. In 2015, at COP21 of the United Nations Framework Convention on Climate Change, 196 countries adopted the Paris Agreement stating that they would limit global temperatures to well below 2°C above pre-industrial levels and would pursue efforts to limit the temperature increase to 1.5°C above pre-industrial levels. The first review of the Paris Agreement was at COP26 in Glasgow with many countries pledging to go to net zero emissions by the middle of the century. But currently these pledges, if fulfilled, will only limit the global average temperature to between 2.4°C and 2.8°C. At COP27 in Egypt the core agreements from the Glasgow Climate Pact were maintained and countries finally agreed to set up a loss and damage facility – although details of who will provide the finance and who can claim are still be to be worked out. This article reviews the key moments in the history of international climate change negotiations and discusses what the key objectives are for future COP meetings.

## Introduction

In 1989 Margaret Thatcher, the then Prime Minster of the UK, gave an address to the United Nations (UN) outlining the science of climate change, the threat it posed to all nations and the actions needed to avert the crisis. She summed up by saying: ‘We should work through this great organisation and its agencies to secure world-wide agreements on ways to cope with the effects of climate change, the thinning of the ozone layer, and the loss of precious species’ [1]. This sentiment was echoed in similar speeches by George Bush Senior, President of the United States, including one in 1992 when he outlined his ‘Clear Skies’ and ‘Global Climate Change’ initiatives [2].

These political speeches occurred because in the 1980s the threat of climate change had finally been recognised. This was in part due to the upturn in the global temperature record, the so called ‘hockey stick’ through the 1980s [3]. This led to the rediscovery of the underpinning science of climate change that had been essentially carried out and settled by the mid-1960s [4]. Our increased knowledge of how changes in atmospheric carbon dioxide (CO_2_) controlled past climate change and significant improvements in supercomputer modelling of our climate system added to our knowledge of anthropogenic climate change [3].

There was also the emergence of global environmental awareness in the 1970s and 1980s. In the 1970s the focus of the environmental movement was inspired and mirrored the concerns of Rachel Carson seminal book *Silent Spring* [5] (1962) with a focus on pollution, pesticides and the destruction of local environments. In the 1980s and early 1990s there was a growing realisation that humanity’s environmental impact was global. This movement was galvanised by the discovery of the ozone hole over Antarctica and the emerging threat of climate change [6]. Seminal reports reinforced this growing global concern such as, the Club of Rome’s 1972 report on *Limits to Growth* and the World Commission on Environment and Development 1987 report *Our Common Future*. By the beginning of the 1990s climate change had become a global issue – even if it was still a highly disputed one [7].

## International conventions and organisations

The Intergovernmental Panel on Climate Change (IPCC) was set up in 1988 and produced its very first science report in 1990 [8]. Two years later, with support from leaders from all around the world, the UN held the Rio Earth Summit – formally called the United Nations Conference on Environment and Development (UNCED) – to help member states cooperate on sustainability and protecting the world’s environment. The Summit was a significant event and led to the Rio Declaration on Environment and Development, the local, national and global sustainability initiative called Agenda 21 and Forest Principles [9]. It was also the first time that parties could sign up to the United Nations Framework Convention on Climate Change (UNFCCC) that the UN set to negotiate limits to global greenhouse gas (GHG) emissions. The conferences also encouraged the parties to join the United Nations Convention to Combat Desertification and the Convention on Biological Diversity.

The UNFCCC came into force on 21 March 1994. As of December 2022, the UNFCCC has 198 parties [10]. The UNFCCC currently operates by ‘agreement by consensus’. Decisions are taken by consensus only because there has been no agreement on the draft rules of procedure and the default is consensus – which of course allows any country to block any decision. One essential principle that is enshrined within UNFCCC process, is ‘common but differentiated responsibilities’ [9]. The latter was born because the UNFCCC acknowledges that different countries have historically emitted varying quantities of GHGs and therefore need to make greater or lesser efforts to reduce their emissions. For example, from 1850 to 2021 the USA emitted over 20% of global cumulative CO_2_ emissions compared with India’s 3.4%. Today, annual per capita emissions of CO_2_ in the United States (US) are about 10 times greater than in India [11]. Many parties within the UNFCCC support the principle of ‘contraction and convergence’ – the idea that every country must reduce its emissions and that all countries must converge on net zero emissions [3]. The importance of a net zero emissions target was agreed in the 2015 Paris Agreement and the seminal IPCC report on Global Warming of 1.5°C published in 2018 showed that to achieve 1.5°C there had to be zero CO_2_ emissions by about 2050 and then negative CO_2_ emissions for the rest of the century [12].

## COP3 Kyoto 1997: the first international treaty

**Figure 1:**
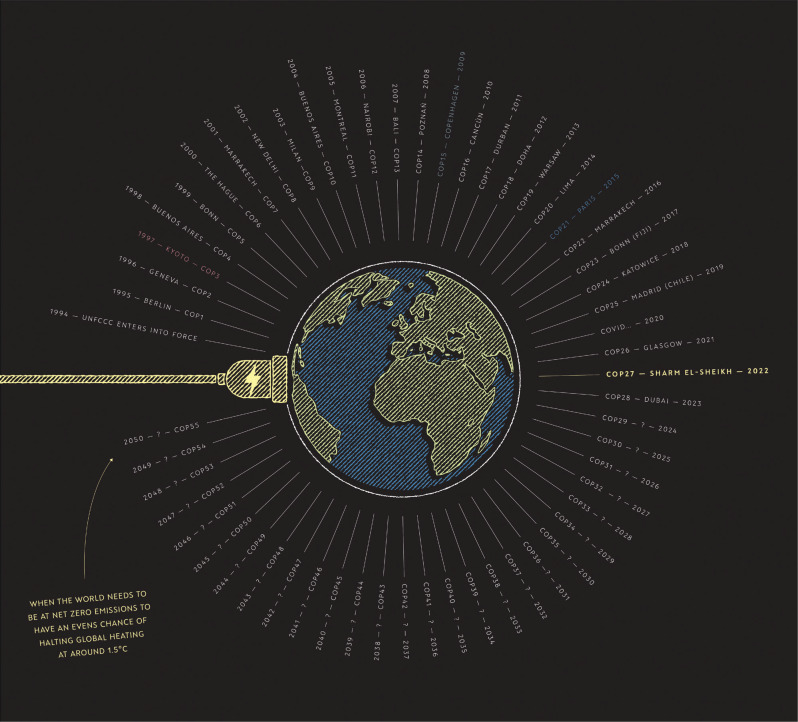
Infographic showing all the UNFCCC COPs from foundation to 2050.

Since the UNFCCC was set up, the nations of the world, ‘the parties’, have met annually at the COP to move negotiations forward (Fig. 1). Only five years after the UNFCCC was created, at COP3 in December 1997, the first international agreement to cut GHG emissions was drawn up, the Kyoto Protocol [9]. This restated the UNFCCC general principles for a worldwide treaty on cutting GHG emissions and, more specifically, that all developed nations would aim to cut their emissions by 5.2% relative to 1990 levels by 2008–2012. Vice-President Al Gore signed the Kyoto Protocol in 1998 for the US, but under the leadership of President George Bush, the US did not ratify the Kyoto Protocol at Bonn in 2001. With the US producing about one-quarter of the world’s CO_2_ pollution at the time, this was a blow for the treaty. The targets set by the Kyoto Protocol were made more flexible during the Bonn meeting to ensure that Japan, Canada and Australia would join. Australia finally made the Kyoto Protocol legally binding in December 2007. The Kyoto Protocol came into force in February 2005, after Russia ratified the treaty, thereby meeting the requirement that at least 55 countries representing more than 55% of the global emissions were participating [9]. To balance out the historic legacy of emissions by developed countries, the treaty did not include developing countries, but it was assumed that developing countries would eventually join the post-2012 agreement.

## COP8–COP13: the technical years

COPs between 2002 and 2005 focused on some of the technical aspects of carbon accounting, carbon trading (the clean development mechanism) and the rules of the Kyoto Protocol. The first COP at which the Kyoto Protocol was legally in force was COP11 in Montreal, Canada, in 2005. After the protocol came into effect, COPs became formally COP/MOPs (meetings of the parties) thus Montreal was also MOP1. Because the US continued to stand outside the Kyoto Protocol, a twin-track approach to the negotiations was adopted, whereby within the COP countries that had ratified the protocol had a series of meetings separate from countries – chiefly the US and Australia, among the major economies – that had not ratified it.

However, leading officials recognised that this arrangement was highly unsatisfactory, and when Yvo de Boer, a Dutch diplomat, was appointed Executive Secretary of the UNFCCC in 2006 he sought the support of the UN Secretary-General to try a different approach, which would look beyond the Kyoto Protocol and try to bring the US on board. This resulted in 2007 in a dramatic COP13 at Bali, where negotiations dragged on far after the official deadline of 6 pm on Friday had passed, and long into the Saturday afternoon, as the US delegation refused to agree to a ‘Bali roadmap’. Finally, after a dramatic intervention on the plenary floor by Kevin Conrad of Papua New Guinea, calling on the US to ‘lead, or get out of the way’, the US delegation led by Paula Dobriansky agreed to join the consensus and the Bali roadmap – which set out a pathway for a unified approach to emissions reduction beyond the Kyoto Protocol – was passed. The Bali roadmap led to the Copenhagen climate summit of 2009, and an attempt at a new model of global climate action.

## COP15 Copenhagen 2009: failure or new dawn?

In 2007, the Nobel Peace Prize was shared, in equal parts, between the IPCC and Al Gore ‘for their efforts to build up and disseminate greater knowledge about human-made climate change, and to lay the foundations for the measures that are needed to counteract such change’. The world had huge expectations for COP15 Copenhagen to deliver in 2009, despite coming just a year after the global financial crash. New quantitative commitments were expected to ensure a post-2012 agreement and seamless transition beyond the Kyoto Protocol. Barack Obama had just become President of the US, raising hopes of a more constructive approach. The European Union (EU) had prepared an unconditional 20% reduction of emissions by 2020 on a 1990 baseline and a conditional target rising to 30% if other developed countries adopted binding targets. Many other developed countries also had something to offer: Norway was willing to reduce emissions by 40% and Japan by 25% from a 1990 baseline; even the US offered a 17% reduction on a 2005 baseline, which was an equivalent drop of 4% compared with 1990. The scene was set, but the Copenhagen conference went horribly wrong: ‘hopenhagen’ became ‘nopenhagen’. First, the Danish government underestimated the interest in the conference, particularly from official non-governmental organisation (NGO) observer organisations, and provided a venue that was too small. In the second week, when all the high-powered national ministers and their entourages arrived, there was insufficient room, meaning many NGO observers were denied access to the conference venue. Second, it was clear that the negotiators were unprepared for the arrival of the ministers and that there was no agreement. This was partly because the Danish pre-conference negotiators who had worked tirelessly to get an agreement were replaced by the Danish Prime Minister’s team during the conference. This led to the leaking of ‘The Danish Text’, subtitled ‘The Copenhagen Agreement’, and the proposed measures to keep average global temperature rise to 2°C above pre-industrial levels [9]. An argument then ensued between developed and developing nations because a brand new text simply appeared in the middle of the conference. China and India took the opportunity to disrupt the negotiations and encouraged other developing countries to accuse developed ones of working opaquely and making an agreement that suited their own interests without seeking consent from developing nations [13]. Lumumba Stanislaus Di-Aping, Chairman of the G77, said, ‘it’s an incredibly imbalanced text intended to subvert, absolutely and completely, two years of negotiations. It does not recognise the proposals and the voice of developing countries’ [14].

The final blow to forging a global agreement based on binding targets came from the US. Barack Obama arrived only two days before the end of the conference. He was fully aware that the US Senate and the House of Representatives would not agree to binding targets. The US negotiators were also fully aware of how China and India were blocking all progress. So Barack Obama convened a meeting with the BASIC (Brazil, South Africa, India and China) countries, excluding all other UN nations, and created the Copenhagen Accord [3]. This recognised the scientific case for keeping temperature rises below 2°C, but did not contain a target baseline, nor commitments for reducing emissions to achieve it. Earlier proposals that would have aimed to limit average temperature rise to 1.5°C and cut CO_2_ emissions by 80% by 2050 were dropped. This was because all mention of a 1.5°C rise was continually blocked by China. The resulting agreement was non-binding and countries could provide their own voluntary targets. It was also made clear that any country that signed up to the Copenhagen Accord was also effectively stepping out of the Kyoto Protocol, hence the US was able to move away from the binding targets of the Kyoto Protocol, which should have been enforced until 2012. A weak voluntary commitment approach was adopted. The Bolivian delegation summed up the way the Copenhagen Accord was reached: ‘anti-democratic, anti-transparent and unacceptable’ [14]. It was also not clear what legal status the Copenhagen Accord had because it was only ‘noted’ by the parties, not adopted, as only 122, subsequently rising to 139 countries, agreed to it [13].

In January 2014, it was revealed that the US Government negotiators had obtained information during the conference by eavesdropping on meetings of other conference delegations. Documents leaked by Edward Snowden suggested the US National Security Agency (NSA) had monitored communications between countries before and during the conference. The leaked documents revealed that the NSA provided US delegates with advance details of the Danish plan to ‘rescue’ the talks should they flounder and laid bare China’s efforts to coordinate its position with India before the conference [15].

At the time Copenhagen was seen as a huge failure. But the Copenhagen Accord laid the foundation for a new style of climate agreement based on country based pledged commitments to an overall global emission reduction target. The Accords were formally adopted under the UN process at COP16 in Cancun the following year and mark the first time that developed and developing countries (non-Annex 1 countries) jointly accepted commitments to take action on GHG emissions. In the case of developed countries, those were emissions cuts; for developing countries, curbs on the future growth of their emissions.

## COP16 Cancun and COP17 Durban: progress is made

At COP16 in Cancun, the UN tried to repair some of the damage of COP15, adopting most of the commitments made in Copenhagen as COP decisions, which formalised them under the UN process. At COP17 in Durban, most countries were expecting a somnolent affair, but Connie Hedegaard, the EU climate commissioner, who in her previous role as Danish Environment Minister had been president of COP15, wanted a different approach. Recognising that the Obama presidency offered a limited opportunity to sign a legally binding agreement, she came to Durban with a plan for a fresh roadmap, to what would eventually become the Paris Agreement. Durban marked a bifurcation among developing countries, which had previously tended to follow the lead provided by China. Many, particularly among the poorest, were increasingly realising that their interests were not aligned, as China’s policies were allowing vast increases in GHG emissions, of which they would bear the brunt. The EU managed to gather a broad coalition of more than 100 such developing countries, and had the tacit approval of developed counterparts such as the US, Japan and Canada, behind its push for a new legally binding agreement. By the scheduled end of the conference, it was clear that two big countries were holding out: China and India. The talks dragged on far beyond their deadline, through Saturday and into the early hours of Sunday, with many countries predicting that the EU would cave, and China and India would succeed in stymying progress. But in the end, no longer able to call on allies among the least developed countries, China and India gave way, and the process towards what became the Paris Agreement was begun.

## COP21 Paris 2015: the Paris Agreement

The failure of COP15 in Copenhagen and its voluntary commitments cast a long shadow over subsequent COP meetings, compounded by the Wikileaks revelation that US aid funding to Bolivia and Ecuador was reduced because of their opposition to the Copenhagen Accord [16]. It took over five years for the negotiations to recover from the difficulties created at Copenhagen. At COP16 in Cancun and COP17 in Durban the UNFCCC negotiations were slowly rejuvenated with the aim of agreeing a new round of negotiations and the underlying principles. There were some important political breakthroughs, including the agreement on the 2°C target, establishment of the Green Climate Fund, the setting of the $100bn a year funding target and the notion of voluntary emission pledges for all – the basic backbone of the Paris Agreement. In parallel, significant progress was made in REDD+ (Reduced Emissions from Deforestation and Forest Degradation), including safeguards for local people [17]. It was not, however, until COP18 in Doha in December 2012 that a second commitment period for the Kyoto Protocol lasting eight years was agreed, to commence from January 2013. This ensured that all Kyoto mechanisms and accounting rules would remain intact, and that parties could review their commitments with a view to increasing ambition. All this laid the foundations for the possibility of a future global climate agreement, which was finally agreed at COP21 in Paris in 2015.

The climate negotiations in Paris 2015 were a huge success in part because of the huge amount preparation work at the previous COPs and at the United Nations Educational, Scientific and Cultural Organisation (UNESCO) ‘Our Common Future Under Climate Change’ international scientific conference held in Paris in July 2015. In May 2015 the Vatican and Pope Francis published a very influential encyclical letter entitled ‘Laudato si’ – on care for our common home’, which was based on up-to-date science [18]. It addressed all humanity and, especially policy makers and called on all countries to protect the Earth, nature and biological diversity and to take ‘swift and unified global action’ to combat climate change, land degradation and to promote economies based on fair distribution of resources and wealth. In addition the French hosts understood the grand game of international negotiation and used every trick in the diplomatic playbook to get countries working together to achieve an agreement signed by all [19]. The Paris Agreement sets a goal to hold temperatures to ‘well below 2°C above pre-industrial levels’ and ‘pursue efforts to limit the temperature increase to 1.5°C above pre-industrial levels’. Paris was a high-stakes game of geopolitical poker. Surprisingly, the least powerful countries did much better than expected. The climate talks were subject to a series of shifting alliances going beyond the usual dichotomy between income-rich global North and income-poor global South countries. Central to this was, first, US–Chinese diplomacy – both agreed to limit emissions in 2014 under the US–China Joint Presidential Statement on Climate Change. Second, a new grouping of countries under the banner of The Climate Vulnerable Forum, including Small Island Developing States, pushed the 1.5°C target higher up the political agenda, so much so that it is mentioned in the key aims of the agreement [19]. Political and civil society support generated from the success of the Paris Agreement created the preconditions for the IPCC to land its special report on global warming of 1.5°C to an attentive global community. Highlighting not just the stark differences between the impact of a 1.5°C and 2°C world [12], the report also modelled pathways of how a 1.5°C world could be achieved: (1) a 45% decline in CO_2_ emissions from 2010 levels by 2030; (2) net zero CO_2_ emissions by 2050; and (3) CO_2_ removal thereafter for the rest of the century. The quicker the world could get to net zero, the less CO_2_ that would need to be sequestered from the atmosphere between 2050 and 2100 [20].

**Figure 2:**
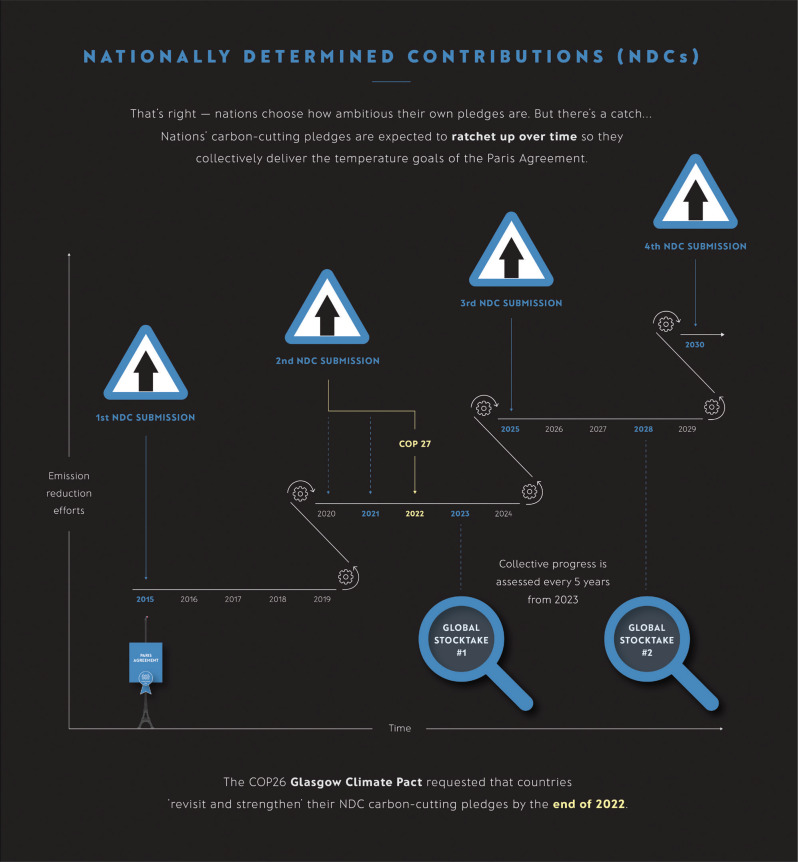
Infographic showing the Paris Agreement (2015) ratchet mechanism to encourage countries to make ever more ambitious emission cut pledges or NDCs.

Crucially, the Paris Agreement was intended to be the start of a process of ratcheting up mitigation ambition (Fig. 2). Collectively, initial pledges were necessary but far from sufficient. Even as late as 2019, assuming all national mitigation pledges were fulfilled, the world would still warm by about 3°C or more [21]. The Paris Agreement also included the Global Stocktake (GST) process with the aim of linking the National Determined Contributions (NDCs) to the overall aim of the Paris Agreement to keeping warming to less than 2°C. The first GST will be COP28 in Dubai in 2023 and will be a major statement on how far we have come towards the aim of the Paris Agreement.

## The role of global environmental social movements

It is possible to construct four main waves of recent environmental social movements. The first was in 1970s and this growing environmental awareness can be traced back to a number of key markers: these include the publication of Rachel Carson’s *Silent Spring* in 1962 [5]; the image of Earth seen from the Moon in 1969; the Club of Rome’s 1972 report on *Limits to Growth*; the Three Mile Island nuclear reactor accident in 1979; the nuclear accident at Chernobyl in 1986; and the *Exxon Valdez* oil spillage in 1989. But this environmental awareness focused mainly on pollution, pesticides and destruction of local environments and seemed to be limited geographically to the specific areas in which they occurred. Many of the key environmental NGOs were formed around these issues, such as Friends of the Earth (1969), Greenpeace (1971) and the World Wildlife Fund (WWF) (1961).

The second wave sprung up in the late 1980s and early 1990s when a growing realisation emerged that humanity’s environmental impact was global. It was the discovery in 1985 by the British Antarctic Survey of depletion of ozone over Antarctica that demonstrated the global connectivity of our environment. The ozone ‘hole’ also had a tangible international cause, the use of chlorofluorocarbons (CFCs), which led to a whole new area of politics: the international management of the environment. There followed a set of key agreements: the 1985 Vienna Convention for the Protection of the Ozone Layer; the 1987 Montreal Protocol on Substances that Deplete the Ozone Layer; and the 1990 London and 1992 Copenhagen Adjustments and Amendments to the Protocol. These have been held up as examples of successful environmental diplomacy. This movement climaxed at the Rio Summit in 1992.

The third wave was in 2008 and 2009, focusing on the hope of a major climate deal at the Copenhagen climate conferences. In the UK it was very successful as political parties looked to ‘rebrand’ and lead to the Climate Change Act being legislated with near unanimous support in 2008 [22]. As we know, Copenhagen ended in failure due to a lack of international leadership, a change in direction driven by the US and China, lobbying by powerful climate change deniers and the global maelstrom of the 2008 global financial crash [3]. For almost 10 years the global environmental movement was held back due to the overwhelming focus on revitalising the global economy. This all changed in 2018.

The fourth wave of the global environmental social movement started in 2018 [23]. In May 2018, Extinction Rebellion was founded in the UK and launched in October 2018 with over 100 academics calling for action on climate change. Using non-violent civil disobedience, Extinction Rebellion aims to compel governments around the world to avoid tipping points in the climate system and stem biodiversity loss to prevent both social and ecological collapse [24]. In November 2018 and April 2019, the group brought Central London to a standstill. Extinction Rebellion quickly spread to at least 60 other cities around the world.

In August 2018, Greta Thunberg – at the age of 15 – began spending her school days outside the Swedish Parliament holding a sign saying *Skolstrejk för klimatet* (school strike for climate) calling for stronger action on climate change. Soon, other students from around the world started similar school strikes on a Friday and called the movement ‘Fridays for Future’ [25]. It has been estimated that, by the beginning of 2020, over 4500 strikes across over 150 countries, involving five million school children had taken place [26]. These strikes were interrupted by the pandemic but have resumed all over the world, including a high-profile ones in Glasgow during the COP26 negotiations.

In 2018 and 2019, three influential IPCC reports were published. First, in 2018, the ‘Special Report on Global Warming of 1.5°C’ was launched, widely regarded as its most important report in its 30-year history. Second, came the ‘Special Report on Climate Change and Land’ and, more specifically, how climate change would impact desertification, land management, food security and terrestrial ecosystems [27]. The third was its ‘Special Report on the Ocean and Cryosphere’ showing the impacts of climate change on the speed of melting ice sheets, mountain glaciers and sea ice, and their implications for sea level rise and marine ecosystems [28].

There is a school of thought that we have now entered a fifth wave of social movement – radical direct action. Borne out of the frustration that many climate campaigners feel from the lack of action by governments and many corporations, direct actions have included: Insulate Britain protestors gluing themselves to motorways; Just Stop Oil protestors throwing soup over famous paintings and then gluing themselves to the frames; protestors letting down the tyres of large sport utility vehicles (SUVs) in cities; and the sabotaging of oil pipelines and refineries [29]. Many activists now see direct action and violence as the only way that government authorities will take note and act [30] – in the US this has been defined as eco-terrorism [31].

## The role of corporations

These new social movements, inspired by the latest science, have compelled some corporations to take a leading role in decarbonising the economy [32]. Microsoft has set the agenda for the technology sector with an ambitious target to become carbon negative by 2030. By 2050 it wants to remove all the carbon pollution from the atmosphere that it and its supply chain has emitted since the founding of the company in 1975. Sky has set the agenda for the media sector, already being carbon neutral – it has pledged its entire value chain will go carbon negative by 2030. BP has also declared that its company operations will be carbon neutral by 2050 by eliminating or offsetting over 415 million tonnes of CO_2_ emissions – although it will still sell oil and natural gas. These companies form part of a group of over 3000 global companies that have pledged to adopt Science Based Targets (https://sciencebasedtargets.org/), meaning, they are all hoping to achieve net zero emissions by the mid-century [33]. But the relationship between corporations and net zero commitments is a difficult one. First a wide range of terms are used by companies, sometimes correctly and sometimes they are used to obfuscate what they are actually doing. For example, net zero, carbon positive, carbon negative, carbon neutral, planet positive, etc. Second, it is not always clear which Scopes the companies are referring to. Scope 1 covers an organisation’s direct emissions from its facilities and activities. Scope 2 cover emissions from energy purchased and used by the organisation. Scope 3 covers indirect emissions such as the supply chain and value chain – which can be over 10 times the emissions of combined Scope 1 and 2. Most companies only refer to Scope 1 and 2 – as these are under their direct control. It is now recognised it is essential for companies to engage supply chains, extraction, production, transportation and consumption if we are to achieve net zero commitments [34].

Third, there is the whole issue of greenwashing – which many organisations and companies have actively engaged in to keep their customers or shareholders happy. The financial think tank Planet Tracker [35] has defined six types of greenwashing which are very helpful when analysing the various claims made by organisations and companies:

1. Greencrowding: hiding in a crowd of other ‘green’ (but vague) do-gooders.
2. Greenlighting: spotlighting a particularly green feature of operations or products to draw attention away from environmentally damaging activities being conducted elsewhere.
3. Greenshifting: implying that the consumer is at fault and shifting the blame.
4. Greenlabelling: where marketers call something green or sustainable, but a closer examination reveals this to be misleading.
5. Greenrinsing: regularly changing environmental, social and governance (ESG) targets before they are achieved.
6. Greenhushing: refers to corporate management teams under-reporting or hiding their sustainability credentials to evade investor scrutiny.

Given this real economic pressure, governments around the world have started to declare climate emergencies and that action must be taken. At the time of publication of this article, over 3250 local governments in at least 40 countries have made climate emergency declarations. Even though between 2020 and 2022 the whole world was focused on dealing with the Covid-19 pandemic, climate change remained a major issue [36]. This was in part due to the large number of extreme weather events report all around the world in 2021 and 2022 [37] and a shift in public perception about the reality and importance of climate change [38].

## COP26 Glasgow 2021: the Glasgow Climate Pact

**Figure 3:**
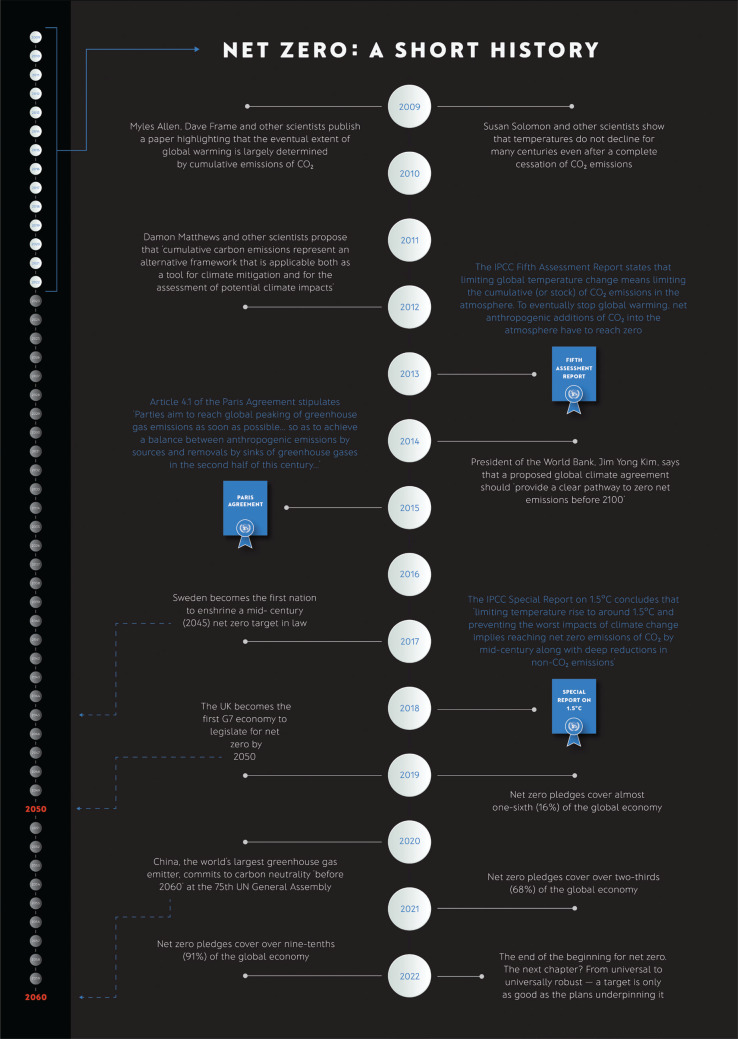
Infographic showing the steps taken towards the net zero emissions target.

This new wave of public global environmental concern meant Copenhagen-esque expectations surrounded COP26 in Glasgow, co-hosted by the UK and Italy and originally scheduled for 2020. But due to Covid-19 enforced restrictions and lockdowns, and the severe impacts on both Italy and Britain, this pivotal meeting was postponed until November 2021 – which provided another year for the background negotiations to take place. COP26 in Glasgow was a different type of negotiation from that of COP21 in Paris, which set the foundations for a new global climate agenda and ambition architecture. Instead, COP26 was critical because it was the third meeting of the parties to the 2015 Paris Agreement (CMA3) and the first-time countries had agreed to raise the ambition of their carbon-cutting pledges, as outlined in the Paris Agreement. Importantly, the focus of COP26 was on ‘net zero’ carbon emission targets – see Fig. 3 [23,32,39].

Despite 2020 and 2021 being dominated by Covid-19, the geopolitical landscape around climate change had shifted seismically before COP26. First, in June 2019, the UK parliament amended the Climate Change Act (2008) to require the government to reduce the UK’s net emissions of GHGs by 100% relative to 1990 levels by 2050 (net zero). Second, the European Commission agreed the EU would reduce its GHG emissions by at least 55% by 2030 from 1990 levels, instead of the 40% cut agreed six years prior. Third, in September 2020, China’s President Xi Jinping announced via video-link to the UN General Assembly that his country would aim to peak CO_2_ emissions before 2030, followed by a long-term target to become carbon neutral by 2060. China, the world’s largest annual emitter of CO_2_ and accountable for around 28% of global GHG emissions, had, up until then, not committed to a long-term emissions goal. Under the Paris Agreement, China had merely pledged to cut the *carbon intensity* of its economy by 60–65% against a 2005 baseline. This announcement followed long and detailed discussions between China and the EU on climate change.

COP26 also marked the re-engagement with the US, the second largest emitter of global GHG emission. President Trump had begun the process of removing the US from the Paris Agreement in 2017, but in accordance with Article 28 of the agreement, a country can only give notice of withdrawal ‘after three years from the date on which [the] Agreement has entered into force’. So, the earliest possible effective withdrawal date by the US was 4 November 2020, one day after the 2020 US presidential election. Newly elected President Biden immediately cancelled the withdrawal and has become a strong advocate of collective international action to deal with climate change.

In many ways COP26 was a small step forward, with little or no back sliding [40]. The Glasgow Climate Pact agreed at the conference includes a strong statement on the necessity of achieving the 1.5°C target – including a renewed call for a 45% reduction in CO_2_ emissions by 2030. The Pact also includes a call to ‘phase down’ coal and remove inefficient fossil fuel subsidies. While the Western press made a lot of fuss over the late switch in language from ‘phase out’ to ‘phase down’ coal (due to pressure from India and China), they missed the fundamental shift in COP terminology: it represented the first time that fossil fuels had ever been mentioned in an international climate agreement [40]. There was also a call in the Pact for new carbon-cutting pledges (NDCs) for COP27 to boost global ambition in the near-term. As stated in the Paris Agreement, NDCs are only required every five years, so the call for new NDCs by COP27 suggested the updates could become annual or biennial to try and accelerate decarbonisation. In Glasgow, the much-maligned and long-awaited Article 6 rulebook was finally finished and signed. This complicated agreement stipulates how countries should monitor and account for their carbon emissions and reductions. It in effect allows countries to sell their emission reductions to others, while at the same time preventing double accounting, so provides a robust framework within which countries and companies can trade carbon [41]. While loopholes exist, finally there was an agreement which everyone could work with [40].

Still, COP26 had notable failures. First, the developed nations failed to honour their 2009 commitment to provide $100 billion per year to help the least developed countries transition from fossil fuels to clean energy. Over a decade after its ratification in 2010 this promise has yet to be fulfilled. Second, COP26 failed to create the Glasgow Loss and Damage facility, whereby the least developed countries could claim compensation from historically high GHG-emitting countries for damage caused by climate change – agreement was blocked by the US and EU on the conference’s last day. Third, countries’ carbon-cutting NDCs are not legally linked to the Paris Agreement 1.5°C pathway – and despite shifts in some countries’ positions, still, even if all pledges are fulfilled the world is still looking at heating of between 2.4°C and 2.8°C by the end of the century [42].

Beyond the Glasgow Climate Pact other international agreements were drawn up on, for example, reducing deforestation, phasing out coal and removing coal finance. An agreement to cut methane emissions by 30% in the next 10 years was secured. There was also an announcement that the US and China would collaborate more closely on climate change throughout the rest of this decade – that is important as, together, they represent 43% of the world’s GHG emissions – but no details have been forthcoming of this collaboration.

## Disruptive influence of the Russian invasion of Ukraine

Geopolitics have altered rapidly in the last few years, with one of key shocks being the Russian invasion of Ukraine. At the time of writing this article there have been about 46,000 deaths, 17 million people have been displaced and 2300 buildings destroyed. In terms of climate change, a mixed response is emerging: the EU is moving away from Russian gas as quickly as possible, having pledged to double the instillation of renewable energy this decade [43]; meanwhile, in the US the Biden administration opened the door to selling new oil and gas drilling leases in the Gulf of Mexico and Alaska to help it ensure self-sufficiency in fossil fuels. It has proposed as many as 11 lease sales over the next five years, including 10 in the Gulf of Mexico and one in the Cook Inlet off the Alaskan coast [44]. Drilling off both the Atlantic and Pacific coasts are not included. However, China, and to a lesser extent India, have been leaping at the opportunity to buy cheap Russian oil, due to Western sanctions on Russian exports. Imports of Russian oil rose by 55% from a year earlier to a record level in May, displacing Saudi Arabia as China’s biggest provider [45]. The Russian invasion meant that oil and gas-producing nations became more influential at COP27, undermining the negotiations [46].

World leaders preoccupied with spiralling energy prices and the escalating cost of living were reluctant to act boldly on fossil fuels. This was reflected in the watered-down text in which the Egyptians slipped in a provision to boost ‘low-emission and renewable energy’ [47], which is a nod to natural gas (cleaner than oil and coal but still a fossil fuel).

Longer term, the invasion of Ukraine has put energy security back on the top of governments’ agendas. For countries with no or little access to domestic fossil fuel reserves, renewables are set to become very attractive – they are already much cheaper to build and maintain than coal fired power stations.

## COP27 Egypt: a missed opportunity

In 2022, two major IPCC reports were published – IPCC Sixth Assessment Report: Working Group II ‘Impacts, adaptation and vulnerability’ [48] and Working Group III ‘Mitigation of climate change’ [49]. Both paint an exceptionally stark future if GHG emissions are not reduced rapidly. Importantly, the reports also demonstrated almost all the solutions required to mitigate and adapt to climate change that already exist – they just need to be scaled up [49]. The possible futures these reports illustrate provided the backdrop to COP27 in Sharm El Sheik, Egypt, billed as the ‘African COP’. The African continent is already experiencing major climate change, including average temperature rises higher than many other parts of the planet and the continent will suffer some of climate change’s most severe future impacts [48].

COP27 turned out to be a rear guard action when negotiators spent most of the time preventing the progress made at Glasgow being watered down or removed. There seemed little enthusiasm from the Egyptian hosts to get any new agreements. At COP26, countries were requested to submit revised carbon-cutting NDCs by COP27 to increase global ambition on mitigating emissions and close the ambition gap necessary to meet the temperature targets of the Paris Agreement. But the COP27 agreement failed to go beyond the 2021 Glasgow Climate Pact’s promise to ‘phase down unabated coal power’ [40], despite the Indian proposal that *all fossil fuels* should be phased out. The text also announced no new targets or commitments, threatening the goal of limiting global temperature rise to 1.5°C, established seven years previously in the Paris Agreement [19]. Instead, there was a request for new country pledges, or NDCs, for COP28 – another year’s delay.

Developing countries entering into COP27 were also hoping for progress on three fronts: climate finance and the delivery of US$100 billion (£84.6 billion) a year as promised in 2009, global decarbonisation and recognition of the responsibility of developed countries to pay for loss and damage. Only one of these was achieved to any degree.

## COP27: loss and damage

Loss and damage refer to the most devastating ravages of extreme weather, so great that no amount of adaptation can help avoid them. Examples include hurricanes and typhoons, the devastating floods that hit Pakistan in the summer of 2022, or the droughts afflicting large areas of Africa in 2022 and 2023. Recovery from such devastation can take years, if it is ever achieved, and the infrastructure of developing countries, services such as health and education, and their chances of improving the lot of their people, can suffer permanent damage.

The world’s poorest countries, which have done least to cause the climate crisis, are most at risk from loss and damage. In the past, some experts characterised loss and damage as a form of compensation or reparations for poor countries, from the rich. However, this was unacceptable to developed and large developing countries, which refused to sign legal agreements potentially leaving them liable for unlimited future costs [50]. Article 8, paragraph 52 in the decision text of the Paris Agreement specifically rejects this characterisation, stating that Article 8 of the Agreement does not involve or provide a basis for any liability or compensation’ [51].

So, the discussion has moved on to loss and damage as a form of rescue and rehabilitation for the countries suffering most, differing from climate finance in that it does not apply to emissions cuts, and addresses broader social and development issues as well as the immediate impacts of extreme weather. For years, little progress has been made on loss and damage discussions, but at COP26 developed countries signalled they would discuss new finance mechanisms for loss and damage. Although COP27 will be viewed as a failure, in the early hours of Sunday morning, well past the Friday deadline, member states agreed to establish such a fund – a win for developing countries. However, who will pay and how this financial assistance will be delivered to help countries such as Pakistan recover from climate disasters remains to be negotiated through 2023.

## Why COP27 failed to deliver

First, the timing of COP27 was unfortunate. Week one occurred during the US midterm elections when much of the world’s media was scrutinising its finely balanced outcome. Week two coincided with the G20 summit in Bali, which further diverted attention and meant many world leaders did not attend. To make matters worse, negotiations stretched into the weekend, just when attention turned to the World Cup and associated controversies in Qatar [52]. This is very different from COP26 when the world remained engaged throughout the summit. The only protests allowed were those sanctioned by the Egyptian security forces within the venue. With media attention already restricted, the limited but important civil society presence at COP27 struggled to keep pressure on the hosts.

Second, there was a lack of leadership. International diplomacy is difficult and takes a huge amount of time, effort and skill. The reason why 2021’s COP26 in Glasgow yielded agreements on deforestation, methane emissions and other issues was partly because the UK and Italian hosts worked hard to build consensus during the extra year provided by the pandemic. Egypt’s presidency of COP27 underestimated this task. When the negotiations carried over to the wee hours of Sunday morning, Egyptian COP27 president, Sameh Shoukry, said: ‘It is really up to the parties [countries] to find consensus’ [53]. This is in stark contrast to COP26, where the president of the conference, Alok Sharma, fought to the bitter end to secure an agreement. Negotiations were only ramped up in the last 48 hours to get an agreement on loss and damage, and even then, some of the larger emitters (China and India) have refused to contribute to the fund.

Third, there is a lack of trust between the developing and developed countries. This is primarily because the US$100 billion promised per year has yet to fully materialise. This is a relatively small amount of money when you consider Qatar reportedly spent $220 billion alone to host the 2022 World Cup [54]. Money to support climate change adaptation has also not been forthcoming. The money is there, the issue is the will to allocate it where it is really needed. And the biggest sticking point was over loss and damage. At COP26, the US, EU and UK, with support from China, blocked the setting up of the Glasgow loss and damage facility, as they did not want to be liable for the effects of climate change. In Egypt, a statement was released at the last minute saying that such a loss and damage fund would be set up after all. It is a step in the right direction and was celebrated by developing nations. But there was no agreement about how large the funding stream would be, who pays, and critically, who controls and manages these funds. Currently, only 10% of climate finance reaches local communities [55] and the new facility will need to address this disconnect. Countries such as China and India pushed back on contributing to those funds. India resisted the inclusion of terms such as ‘current high emitters’ in the text as it expects historically high emitters to contribute to the funds. This may have also been the case for China 30 years ago. But now China’s historic emissions are nearly as high as the EUs, so it points to per capita emissions and has restated its status as a developing country.

## Convention on Biological Diversity COP15

In December 2022 the Global Biodiversity Framework (GBF) was agreed and adopted at the Convention on Biological Diversity COP15. For many it is still strange that the COP for Climate Change and Biodiversity are run separately, with the former getting a lot more press and recognition. At COP15 a large number of targets were set including by 2030: protecting 30% of Earth’s lands, oceans, coastal areas, inland waters; restoration completed or underway on at least 30% of degraded terrestrial, inland waters and coastal and marine ecosystems; reducing to near zero the loss of areas of high biodiversity importance, including ecosystems of high ecological integrity; and cutting global food waste in half and significantly reducing over-consumption and waste generation. COP15 made it very clear that climate change is having a huge negative impact on biodiversity. It also recognised that biodiversity and ecosystems, through nature-based solutions such as the protection and expansion of rainforests, mangrove forests, wetlands, peatlands and coral reefs, is an important way to remove carbon from the atmosphere and helps create climate resilience. Protecting the world’s ecosystems will help safeguard the world’s climate.

## Conclusion

**Figure 4:**
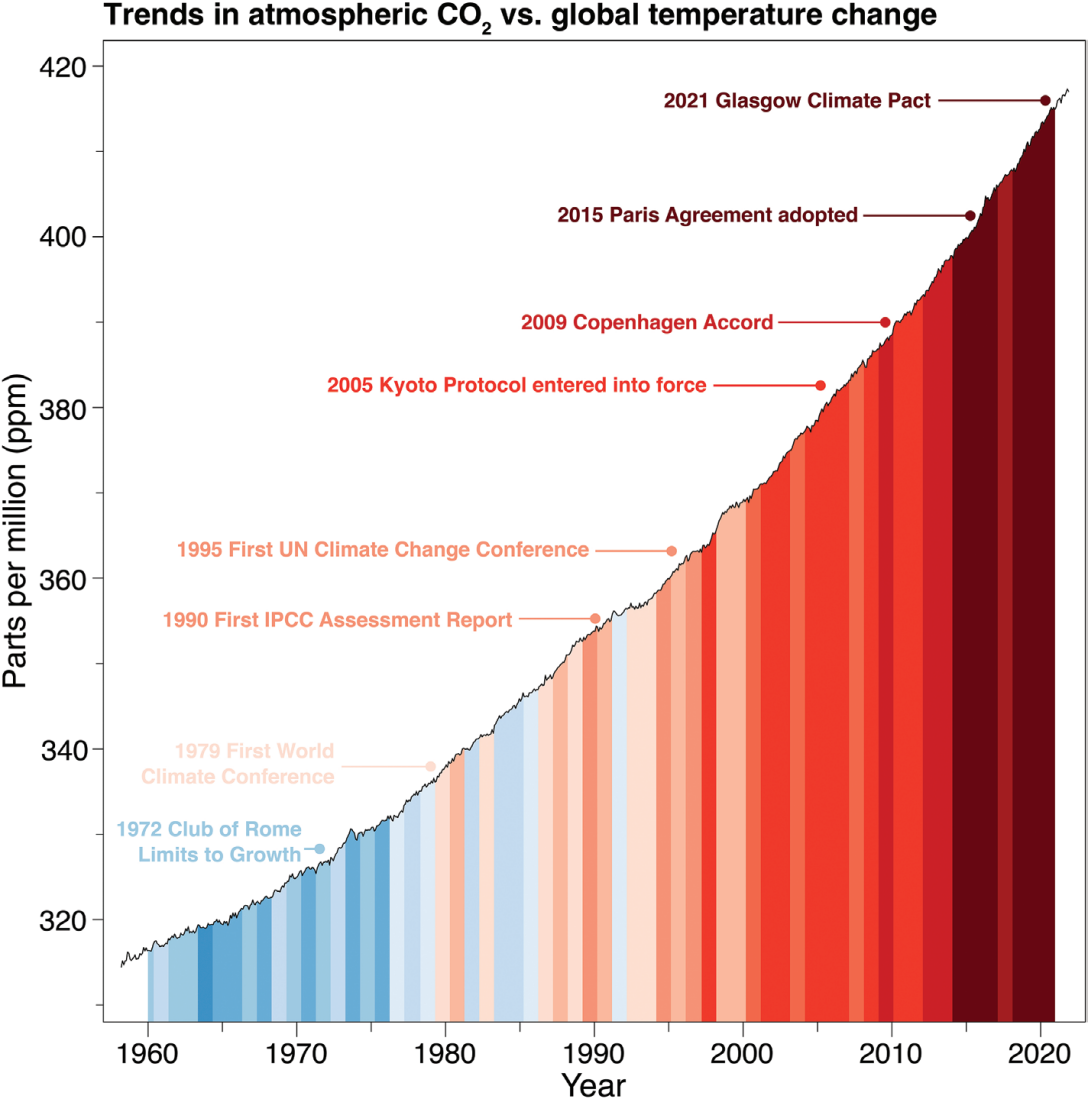
CO_2_ levels and global temperature strips compared to key climate change meetings.

In the last 30 years the amount of human-emitted CO_2_ has doubled, representing a collective failure of the world’s leaders to rise to Margaret Thatcher’s 1989 global call to action (Fig. 4). However, the climate negotiations and the resultant changes in the global economy mean that predicted levels of global warming above 4°C this century are highly unlikely. If all the NDCs made at the Glasgow COP26 meeting are fulfilled then global warming would be kept to between 2.4°C and 2.8°C. Despite this progress we can still view the negotiations as a political failure, as climate change impacts are increasing and we are nowhere near keeping global warming to just 1.5°C above pre-industrial levels. But consider this huge change in ambition - in 1997, COP3 and the resulting Kyoto Protocol aimed for developed countries to cut emissions by only 5.2% relative to their 1990 levels [56], twenty-five years later at COP27, negotiations are aimed to get all countries to agree to be ‘net zero’ emissions by the middle of this century.

We will have to wait and see what effect the Russian invasion of Ukraine has on the long-term demand for fossil fuels, but it is becoming clearer that energy security is synonymous with energy decarbonisation. An interesting geopolitical back drop for COP28 in Dubai is emerging, first is the clear conflict between petrochemical states and actors and the need to reduce global emissions. This conflict is also within states, for example the host of COP28 the UAE are aiming to be net zero by 2050 but want to double their oil and gas exports by 2030. COP28 will focus on adaptation, giving it equal billing to mitigation, and there is a push to get more ambitious NDCs out of obstinate countries. There is also hope of securing details of the loss and damage facility announced at COP27.

Few underestimate how difficult, and important, it is to negotiate GHG reductions at the multilateral level but securing progress has always been painstakingly slow. The avoidance of extreme global warming is a success that should be acknowledge though we still have a long way to go to avoid major impacts of climate change. We should also acknowledge that currently 92% of the world GDP and 88% of global GHG emissions are under a net zero emissions pledge (https://zerotracker.net/) – now all we have to do is ensure each country makes good on their pledges and also increases their ambition.

## Data Availability

All data generated or analysed during this study are included in this published article (and its supplementary information files).

## References

[1] Margaret Thatcher Foundation (1989). Speech to United Nations General Assembly (Global Environment).

[2] Bell A (2021). Our biggest experiment: an epic history of the climate crisis.

[3] Maslin MA (2021). Climate change: a very short introduction.

[4] Weart SR (2008). The discovery of global warming, new histories of science, technology, and medicine.

[5] Carson R (1962). Silent spring.

[6] Corfee-Morlot J, Maslin M, Burgess J (2007). Global warming in the public sphere. Philos Trans A Math Phys Eng Sci.

[7] Oreskes N, Conway EM (2012). Merchants of doubt: how a handful of scientists obscured the truth on issues from tobacco smoke to global warming.

[8] Intergovernmental Panel on Climate Change (IPCC) First assessment report overview and policymaker summaries.

[9] Gupta J (2014). The history of global climate governance.

[10] United Nations Framework Convention on Climate Change (UNFCCC) (2022). Status of ratification of the convention.

[11] Evans S (2021). Analysis: which countries are historically responsible for climate change?. Carbon Brief.

[12] Intergovernmental Panel on Climate Change (IPCC) (2018). Special report: global warming of 1.5°C.

[13] Byrne A, Maslin MA (2015). Negotiating failure: understanding the geopolitics of climate change: review essay. Geogr J.

[14] Goldenberg S, Helm T, Vidal J (2009). Copenhagen: the key players and how they rated. The Guardian.

[15] Vidal J, Goldenberg S (2014). Snowden revelations of NSA spying on Copenhagen climate talks spark anger. The Guardian.

[16] Carrington D (2010). WikiLeaks cables reveal how US manipulated climate accord. The Guardian.

[17] FAO (2022). The Food and Agriculture Organization of the United Nations. https://www.fao.org/redd/overview/en/.

[18] Vatican (2015). Encyclical letter: Laudato si’ of the Holy Father Francis: on care for our common home.

[19] Lewis SL (2015). Five things you need to know about the Paris climate deal. The Conversation.

[20] Goodall C (2020). What we need to do now: for a zero carbon future.

[21] Maslin MA (2019). Climate change: essential knowledge for developing holistic solutions to our climate crisis. Emerg Top Life Sci.

[22] Bryne AJG (2019). The origins, design and implementation of the UK Climate Change Act 2008. PhD thesis.

[23] Figueres C, Rivett-Carnac T (2020). The future we choose: surviving the climate crisis.

[24] Lewis SL, Maslin MA (2018). The human planet: how humans caused the anthropocene.

[25] Thunberg G (2019). No one is too small to make a difference.

[26] Fridays for Future (2022). Strike statistics.

[27] Intergovernmental Panel on Climate Change (IPCC) (2019). Special report: climate change and land. https://www.ipcc.ch/srccl/.

[28] Intergovernmental Panel on Climate Change (IPCC) (2019). Special report: ocean and cryosphere in a changing climate. https://www.ipcc.ch/srocc/.

[29] Gayle D (2022). Just Stop Oil campaigners glue themselves to Da Vinci copy in Royal Academy. The Guardian.

[30] Malm A (2021). How to blow up a pipeline: learning to fight in a world on fire.

[31] FBI (2002). Federal Bureau of Investigation – Congressional Testimony (archive.org).

[32] Hawken P (2018). Drawdown: the most comprehensive plan ever proposed to reverse global warming.

[33] Carbon Disclosure Project (CDP) (2019). Climate change report 2018.

[34] Lloyd SM, Hadziosmanovic M, Rahimi K, Bhatia P (2022). Trends show companies are ready for Scope 3 reporting with US Climate Disclosure Rule. World Resources Institute.

[35] Planet Tracker (2023). Greenwashing is becoming increasingly sophisticated.

[36] Jones PJS, Maslin M (2020). Governance of the global environmental crisis post-COVID-19.

[37] World Meteorological Organisation (WMO) 2022 United in science report.

[38] UNDP (2021). Peoples climate vote report.

[39] Mann M (2021). The new climate war: the fight to take back our planet..

[40] Lewis SL, Maslin MA (2021). Five things you need to know about the Glasgow climate pact. The Conversation.

[41] World Bank (2022). What you need to know about Article 6 of the Paris Agreement.

[42] Climate Action Tracker (2022). Temperatures.

[43] Hnidyi V, Spicer J (2022). Russia, Ukraine to sign deal on restarting grain exports, Turkey says. Reuters.

[44] Natter A (2022). Biden’s offshore drilling plan could bring new lease sales. Bloomberg.

[45] Hoskins P (2022). Ukraine war: Russia becomes China’s biggest oil supplier. BBC News.

[46] Millan L (2022). Middle East oil giants assert themselves in climate politics. Bloomberg.

[47] UNFCCC (2022). Sharm el-Sheikh implementation plan.

[48] IPCC. Climate change 2022: impacts, adaptation and vulnerability (2022). Contribution of Working Group II to the Sixth Assessment Report of the Intergovernmental Panel on Climate Change.

[49] IPCC. Climate change 2022: mitigation of climate change (2022). Contribution of Working Group III to the Sixth Assessment Report of the Intergovernmental Panel on Climate Change.

[50] Calliari E, Serdeczny O, Vanhala L (2020). Making sense of the politics in the climate change loss & damage debate. Glob Environ Change.

[51] Basu J (2022). COP27: Sameh Shoukry urges countries to reinforce UNFCCC credibility as talks drag on. DownToEarth.

[52] Bhattacharya A (2022). Qatar’s $200 billion splurge will be hard to justify when the World Cup ends. Yahoo! Finance.

[53] International Institute for Environment and Development (IIED) (2019). Climate finance not reaching the local level. IIED.

[54] Maslin MA (2020). The road from Rio to Glasgow: a short history of the climate change negotiations.. Scotti Geogr J.

[55] Leggett JK (2018). The winning of the carbon war: power and politics on the front lines of climate and clean energy.

[56] BBC Sport (2022). Qatar’s promise.

[57] Bodansky D (2021). Audiovisual Library of International Law.

